# Challenges of Conducting a Feasibility Pilot Randomized Controlled Trial During COVID-19 Restrictions: Rivaroxaban Versus Acetyl Salicylic Acid in Patients with Multiple Myeloma on Lenalidomide-Based Therapy (RithMM)

**DOI:** 10.3390/curroncol33100633

**Published:** 2026-10-20

**Authors:** Matthew Strelau, Gregoire Le Gal, Arleigh McCurdy, Sudeep Shivakumar, Jared C. Wootten, Maisam Abouzeenni, Chai W. Phua, Selay Lam, Alejandro Lazo-Langner, Mohamed Y. Abouzeenni, Martha Louzada

**Affiliations:** 1 Division of Hematology, Department of Oncology, London Health Sciences Centre, University of Western Ontario, London, ON N6A 5W9, Canada; matthew.strelau@mytwu.ca (M.S.); j.wootten@ucl.ac.uk (J.C.W.); maisam.abouzeenni@lhsc.on.ca (M.A.); chai.phua@lhsc.on.ca (C.W.P.); selay.lam@lhsc.on.ca (S.L.); alejandro.lazolangner@lhsc.on.ca (A.L.-L.); mohamed.abouzeenni@lhsc.on.ca (M.Y.A.); 2 Department of Medicine, Ottawa Hospital Research Institute, University of Ottawa, Ottawa, ON K1H 8L6, Canada; glegal@ohri.ca (G.L.G.); amccurdy@toh.ca (A.M.); 3 Department of Medicine, Dalhousie University, Nova Scotia Health Authority, Halifax, NS B3H 4R2, Canada; sudeep.shivakumar@nshealth.ca; 4 Department of Epidemiology and Biostatistics, University of Western Ontario, London, ON N6A 3K7, Canada

**Keywords:** multiple myeloma, venous thromboembolism, anticoagulation, rivaroxaban, ASA, thromboprophylaxis, lenalidomide, dexamethasone

## Abstract

Venous thromboembolism is a serious medical complication with an increased risk in patients with multiple myeloma undergoing combination treatment regimens. Acetylsalicylic acid is routinely used as a method of thromboprophylaxis in multiple myeloma patients treated with lenalidomide, and rivaroxaban has been assessed in small-scale studies in multiple myeloma patients treated with immunomodulatory drugs; yet, no study has compared the two methods. In this pilot, we asked if a full-scale trial would be feasible to assess the benefits of rivaroxaban over acetylsalicylic acid as a thromboprophylactic approach. This pilot recruited patients with multiple myeloma on lenalidomide-based therapy and randomized them to either low-dose acetylsalicylic acid or rivaroxaban, then monitored bleeding and thrombotic events throughout lenalidomide–dexamethasone treatment. A total of 55 patients were recruited across three Canadian centres, with two venous thromboembolic events and two bleeding events, all in the acetylsalicylic acid arm. We conducted a post hoc analysis of active recruitment time as a definition of recruitment feasibility, as we did not achieve our pre-defined feasibility goals. Despite the challenges in enrollment, possibly due to social restrictions during the COVID-19 pandemic, we maintain that a full-scale trial is feasible and offers insight into thromboprophylactic management in this population.

## Simple Summary

Multiple myeloma patients are highly vulnerable to serious blood clots, a complication that significantly increases their risk of death. Because current guidelines lack clear recommendations on optimal preventative care, this study aimed to compare two common blood thinners. We assessed whether a future clinical study could determine if these drugs could safely prevent clot formation in patients receiving standard cancer treatments. Due to significant obstacles from public health shutdowns during the COVID-19 pandemic, we did not successfully reach our feasibility goals, but still maintain the potential for a full-scale trial. This study paves the way for future research that will guide which blood thinner provides the best safety profile and effectiveness for improving care in this vulnerable patient population.

## 1. Introduction

Venous thromboembolism (VTE), including deep vein thrombosis (DVT) and pulmonary embolism (PE), is the third leading cause of cardiovascular mortality in North America, with an incidence of 100–150 per 100,000 people per year [1,2]. Patients diagnosed with hematological malignancies, such as multiple myeloma (MM), face an increased risk of VTE relative to the general population due to the inflammatory response to MM, as well as combination treatments with immunomodulatory drugs, chemotherapies, and steroids [3]. The incidence of VTE in patients with MM varies greatly, often between 6% and 26%, depending on the disease stage, anti-myeloma treatment, age, and cardiovascular risk factors [4,5,6].

Little is known about arterial thromboembolism (ATE) in the context of MM treatment; however, ATE has been increasingly recognized as a complication of MM and MM treatment, including cerebrovascular disease and coronary arterial disease [7,8,9]. Several studies suggest that the incidence of either ATE or VTE is highest in the initial six months of combination therapy with immunomodulatory drugs, such as lenalidomide [10,11,12,13]. Lenalidomide is the current standard of care medication in combination therapy for both newly diagnosed MM (NDMM) and relapsed and refractory MM (RRMM) patients. A systematic review identified low-dose acetylsalicylic acid (ASA) as the most commonly used method of thromboprophylaxis in patients diagnosed with MM on lenalidomide-based therapy, regardless of VTE risk estimated by the International Myeloma Working Group (IMWG) criteria [14]. In addition to lenalidomide, the corticosteroid dexamethasone is a widely used medication in combination therapy for MM and may influence the risk of VTE. In patients on lenalidomide and low-dose dexamethasone (≤20 mg weekly), the average risk for VTE was 10% [95% CI: 7.18–14.58], whereas those on lenalidomide and high-dose dexamethasone (≥40 mg weekly) had up to 26% [95% CI: 20.9–33.2] [RR = 2.5 (95% CI: 1.68–3.96), *p* < 0.0001] [14]. This suggests a strong association between steroid dose and VTE risk. Additionally, the use of anticoagulants can reduce the risk of VTE. The incidence of VTE in patients on low molecular weight heparin (LMWH) was significantly lower at 1.4% [95% CI: 0.48–4.09] [14]. This was also investigated by Larocca and colleagues in an open-label randomized sub-study of myeloma patients on lenalidomide combination therapy to assess their risk of VTE and ATE [13]. Three hundred forty-two myeloma patients with no clinical indication or risk of antiplatelet or anticoagulant therapy were randomized to either daily acetylsalicylic acid (ASA) 100 mg (n = 176) or enoxaparin 40 mg (n = 166). Patients on ASA were shown to have a rate of VTE of 2.27%, and those on enoxaparin 1.2% [13], and there were no cases of ATE reported [13]. Other medications, such as direct oral anticoagulants (DOACs), have been used for the treatment of VTE and stroke prevention in atrial fibrillation in the past 15 years. DOACs have also been evaluated in small cohort studies in patients with MM and offer an alternative to antithrombotic approaches [15,16,17]. The evidence has suggested that VTE is reduced in patients with MM regularly taking DOAC medications compared to ASA [16,17]. One such DOAC is rivaroxaban, which reduces VTE by inhibiting Factor Xa in the coagulation cascade. Rivaroxaban offers several advantages amongst the other DOACs; it is easy to administer—with one pill taken daily, it has a high oral bioavailability of 80% with peak plasma concentrations achieved around 2.5–4 h, and it is also more cost-effective than LMWH (approximately 1.3 CAD per pill, whereas LMWH is 13 CAD per injection) [18]. Despite these advantages, current oncology guidelines have yet to provide specific recommendations for the use of DOACs for primary prophylaxis in patients with MM on the lenalidomide-based therapies [10,19]. Given the lack of evidence, it is important to address the gap in risk assessment of ATE, and VTE in patients with MM on lenalidomide and dexamethasone backbone (Len-Dex). This study assesses the feasibility of conducting a full-scale trial to assess rivaroxaban compared to ASA for improvement of thromboembolism outcomes in patients with multiple myeloma on lenalidomide-based therapy (RithMM).

## 2. Materials and Methods

### 2.1. Study Design and Setting

This study was a feasibility pilot, investigator-driven, Canada-based, multi-centre, open-label randomized controlled trial that occurred from January 2019 to February 2023 and was designed to evaluate the feasibility of a clinical trial assessing the efficacy and safety of rivaroxaban 10 mg daily compared to ASA 81 mg daily for the prevention of VTE or ATE in patients with multiple myeloma on Len/Dex-based therapy. Patients with NDMM or RRMM receiving lenalidomide and dexamethasone with or without combination with other anti-myeloma drugs were assessed for eligibility to be enrolled in the study. We sought to enroll 86 adults with multiple myeloma at three Canadian sites: London Health Sciences Centre, Ottawa Hospital Research Institute, and Nova Scotia Health Authority. Participants were monitored for adverse events and efficacy through physical exams, blood tests, and blood pressure measurements. This study was approved by the Institutional Review Board and was registered through ClinicalTrials.gov (NCT03428373) in 2016, with updates made in October 2026.

The trial was conducted in accordance with the International Council for Harmonisation Good Clinical Practice guidelines, the principles originating from the Declaration of Helsinki, and site-specific regulations. All participants were provided with written informed consent.

### 2.2. Participants

We included patients between January 2019 and February 2023 meeting the following criteria: (1) diagnosis of MM, (2) scheduled to start on Len/Dex therapy, (3) aged 18 or older, and (4) able to provide written informed consent. We excluded patients who had (1) platelet count of ≤50 × 10^9^/L and a Creatinine Clearance (CrCl) ≤ 15 mL/min, (2) severe liver disease in the last 6 months, (3) major bleeding event(s) within 3 months prior to starting Len/Dex, (4) history of antiphospholipid antibody syndrome, (5) history of malignancy other than MM within 2 years prior to randomization (or with evidence of residual disease (basal cell or squamous carcinoma were not excluded)), (6) gastric or duodenal ulcer within 2 years, (7) plasma cell leukemia; systemic amyloidosis, (8) therapeutic anticoagulation for treatment of VTE or ATE, stroke prevention in non-valvular atrial fibrillation (those with a history of VTE who were not on anticoagulants were not excluded), (9) antiplatelet agents due to an absolute indication, (10) concomitant systemic treatment with strong inhibitors of both CYP 3A4 and P-gp, (11) single agent lenalidomide, (12) life expectancy less than 3 months as determined by the investigator, (13) unstable medical or psychological condition that would interfere with participation as determined by the investigator, (14) uncontrolled cardiovascular disease within 6 months prior to enrollment, (15) uncontrolled/poorly controlled diabetes or renal disease, (16) major surgery within 2 weeks of randomization, (17) allergies, hypersensitivity or intolerance to any of the study drugs, (18) not able or willing to give consent.

### 2.3. Sample Size Justification

The recruitment target for the RithMM pilot was calculated from historical averages of patient volumes in London, Ontario, who were treated with lenalidomide-based therapy. From January 2015 to December 2015, 60 myeloma patients were started on lenalidomide treatment in London, Canada. This is equivalent to an average of 5 patients per month. Of those 60 patients, 12 (20%) started on single-agent lenalidomide, decreasing the number of potentially eligible RithMM trial participants to approximately 48 per year, or 4 patients per month. If we consider that approximately 20% (n = 10) of patients are not eligible or not willing to participate in the trial, we estimate 38 eligible patients per year, or about 3 patients per month at London Health Sciences Centre. Accounting for potential differences in accrual performance and potential loss to follow-up, the reasonable target average recruitment rate would be a minimum of 1 to 2 patients per month, per centre, for 12 months. For this study, per protocol, we set an enrollment goal of 86 total patients in 12 months between the three participating sites (London, Ottawa, and Halifax). However, due to the unanticipated COVID-19 restrictions, we normalized recruitment calendar time to *active recruitment time* as an exploratory analysis. *Active recruitment time* was defined as the interval from the first participant’s enrollment to the onset of a period of more than six consecutive months without a new participant providing informed consent. *Inactive recruitment* was defined as a period of more than six consecutive months during which no new participants provided informed consent. The principal investigator (Louzada) adjudicated the occurrence of such periods.

For a full-scale clinical trial, we used a power equation that gives a required number of events to yield 90% power with a 0.05 (2-sided) alpha significance. A sample size of 362 participants (181 in each group) is required to provide a statistical power of 90% utilizing the Fleiss method without continuity correction, considering that patients on ASA will have a 6-month-cumulative 10% TE rate and those on rivaroxaban, 2%. The respective incidences are not based on the results of the pilot study, which was not powered to answer this question, but on previously published data, including our systematic review and retrospective cohort study [8,14,20,21,22,23]. The estimated proportion of patients who will experience attrition from the study is established at 10%. Therefore, accounting for statistical power and loss to follow-up, a total of 398 (199 in each group) participants, from seven participating sites (57 participants per site), is required to demonstrate that rivaroxaban is superior to ASA.

### 2.4. Intervention

Study data were collected, managed, and randomized using REDCap electronic data capture tools hosted at London Health Sciences Centre [24,25]. Randomization was stratified according to site and to the lenalidomide line of therapy (first line or second line). All patients enrolled in the study were randomized to one of the following arms: 10 mg of rivaroxaban orally once daily starting at the time of randomization for 6 months of Len/Dex-based therapy, or 81 mg of ASA orally once daily starting at the time of randomization for 6 months of Len/Dex start. Anticoagulant treatment had started at the same time as Len/Dex, and patients were followed for 6 months, or until one of the following occurred: 4 weeks after stopping lenalidomide, they withdrew from the study, the patient was lost to follow-up, or they died.

We collected data on vitals and blood work, including creatinine clearance to assess renal function, as well as the biomarkers D-dimer, C-reactive protein, beta2 microglobulin, and LDH to assess their potential validity as predictors of hypercoagulability. This collection was used to assess the feasibility of participating sites to collect study-specific blood work with every participant follow-up (baseline and 3 follow-up visits). Data collection and participation adherence feasibility was considered successful when 90% of events were collected, in which case this evaluation method and strategy would be considered suitable for implementation in a full-scale trial.

The primary outcome of the RithMM pilot was the feasibility of accrual of participants within 12 months per included site. The study was to be considered feasible if 86 patients across 3 Canadian sites (London, Ottawa, Halifax) could be successfully enrolled within 12 months of each site’s activation, with an estimated enrollment rate of 1 to 2 patients per site per month. If feasible, the pilot study results would drive the methodology for a full-scale, multi-centre RithMM clinical trial. Secondary feasibility outcomes were dropout rates and medication compliance. We estimate that a dropout rate of 10% or less is reasonable and we estimate a medication compliance of 90% as optimal to move forward with the full trial.

We assessed secondary efficacy outcomes using withdrawal or loss to follow-up, the rate of compliance with study drug administration, and the length of time to obtain ethics approval and contracts for study sites. Additional clinical outcomes include the TE incidence rate, major bleeding and clinically relevant non-major bleeding rates, minor bleeding rates, all-cause mortality, and a subgroup analysis stratifying patients into low- and high-risk of TE (if appropriate, given this study is not powered to show a statistically meaningful difference for clinical outcomes); and assessment of the relevance of measuring myeloma biomarkers and their potential association with TE risk. TE was assessed during visits through symptoms such as, but not limited to, leg tenderness, swelling, and/or erythema suspicious for deep venous thrombosis; weakness in arms or legs or slurred speech or confusion, facial droop, suspicious for stroke; acute onset of shortness of breath, pleuritic chest pain, fever or chills or hemoptysis, suspicious for pulmonary embolism or acute coronary syndrome; and other symptoms of thrombosis. Any hospital encounters associated with any of the above-mentioned complaints were captured and any tests or procedures done were recorded (e.g., echocardiogram, ECG, CT scan, MRI).

We also assessed the association between biomarkers related to inflammation and hypercoagulability (D-Dimer, β-2 microglobulin, CRP, and LDH) and the incidence of TE. Biomarkers had to be collected at baseline, at 1-month, 3-month follow-up visits and at the end of the study visit. It is well established that D-Dimer levels are elevated at VTE diagnosis [26]. It is unknown whether there is a direct correlation between D-Dimer levels and the diagnosis of VTE or ATE in MM. Additionally, β2-microglobulin and LDH are prognostic markers in MM and are routinely monitored in clinical practice. It is often observed that higher measured β2-microglobulin and LDH are associated with a decrease in overall survival [27,28,29]. However, it remains unknown whether there is a correlation between these biomarkers’ concentrations and the risk of TE. Lastly, we investigated CRP, as CRP is a universal inflammatory marker linked to poorer prognosis [30,31]. Inflammation is present in MM, VTE and ATE. However, it is currently unknown whether CRP is predictive of TE in the context of patients with MM on Len/Dex. Safety efficacy outcomes were included to describe and report the incidence of major bleeding and clinically significant non-major bleeding (CRNMB) in each study arm.

### 2.5. Statistical Analysis

Feasibility indicators were calculated as simple frequencies and proportions. We measured the recruitment rate monthly for each centre by measuring the first date of participant consent for the respective centre and the date of the final participant consent for the respective centre. Detailed screening and enrollment logs were completed at each participating centre. Screening logs included reasons for eligibility and ineligibility. We collected detailed data about recruitment rates, non-compliance, withdrawal/loss to follow-up, and event outcome rates. We planned an interim analysis to be completed in 6 months for each site, and if 40 ± 3 patients were enrolled, the interim performance of the recruitment rate would be feasible.

This study was not powered to detect statistically significant differences in clinical or laboratory outcomes; therefore, these outcomes will be presented in the manuscript as descriptive statistics only.

The clinical assessment of the study followed a primary intention-to-treat analysis where patients must take at least 1 dose of the study drug. We calculated the 6-month incidence of symptomatic acute VTE. For ATE, we calculated the 6-month incidence of myocardial infarction and ischemic stroke and major + CRNMB. Major bleeding and CRNMB were defined as by Schulman & Kearon 2005 and Kaatz et al., 2015 [32,33]. Means, medians, and frequency distributions were calculated for continuous variables. Assessment of biomarkers included compliance with blood work and exploratory analysis of TE risk according to differences in baseline and end-of-study levels. Values and percentages were reported for binary and categorical variables. Differences between subgroups of interest were examined using the Wilcoxon Rank Sum for continuous variables. All tests were two-tailed, and a *p* < 0.05 was assumed to represent statistical significance. All analyses were performed using STATA 18.5 BE and RStudio 2026.07.1+147. Considering this is a pilot study, analyses were conducted using all available data at each time point, with the number of observations varying according to data availability.

## 3. Results

### 3.1. Recruitment and Retention

**Figure 1:**
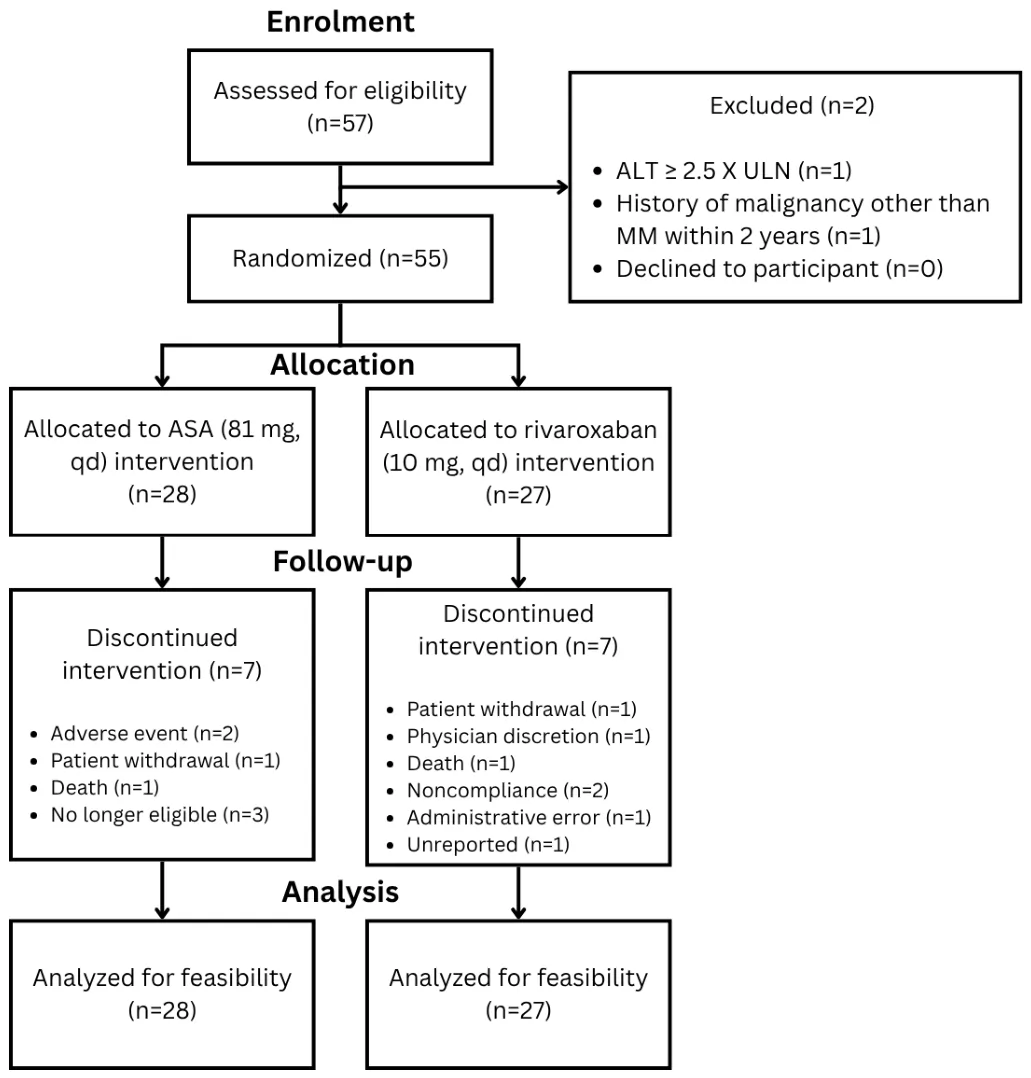
Flow diagram of patients enrolled in the trial. ALT, alanine aminotransferase; ULN, upper limit of normal; MM, multiple myeloma; and ASA, acetylsalicylic acid.

Potential patients to participate in the study were approached between January 2019 and February 2023. This pilot was intended to close once the final enrolled patient completed their 6 months of treatment, which was expected to occur sooner than 2023. However, the prolonged period during which the study remained open is the consequence of repeated COVID-19 public health social restrictions between 2020 and 2021, as well as institutional policies that prioritized essential medical care over non-essential research activities. The combination of federal, provincial, and site-specific policies created unexpected and indirect obstacles to reaching the projected recruitment targets at each centre. Overall, a total of 55 patients with MM were enrolled and successfully screened (Figure 1); see Section 3.2 for details on the feasibility outcomes. Of these, 27 participants were randomized to the rivaroxaban arm, and 28 participants were assigned to the ASA arm. There were 34 (61.8%) males and 21 females (38.2%), with a median age at baseline of 74 and a mean age of 72.6. Three (5.5%) participants, all from the ASA arm, had a previous history of gastrointestinal bleeding and ten (18.18%) had a history of cardiovascular disease. Bleeding risks were determined through the Bleeding Index Score [34]. For those on ASA, 4 (14.3%) were ranked as low risk, 21 (75%) as intermediate and 3 (10.7%) as high risk. The rivaroxaban group had 7 (25.9%), 18 (66.7%), and 2 (7.4%) patients, respectively, as shown in Table 1.

The participants’ compliance with the anticoagulant regimens at each timepoint is summarized in Table 2. Most participants were fully adherent to the corresponding anticoagulant regimen at each timepoint: 87.3%, 70.9%, and 69.1% at 1 month, 3 months, and 6 months, respectively. Attrition occurred in 25.5% of participants, most commonly due to a change in participant eligibility (n = 3, 5.4%). A total of 43 participants (78.2%) attended the final study visit at 6 months, including two participants that stopped the drug intervention. Anticoagulant compliance data, a reflection of adherence up to and including a participant’s final documented visit, were available at 6 months for 42 of these 43 participants: 38 (69.1%) reported 100% compliance, 3 (5.5%) reported 99–75% compliance, and 1 (1.8%) reported 0–24% compliance. One participant who attended the final visit did not have compliance data recorded.

### 3.2. Feasibility Outcomes

**Figure 2:**
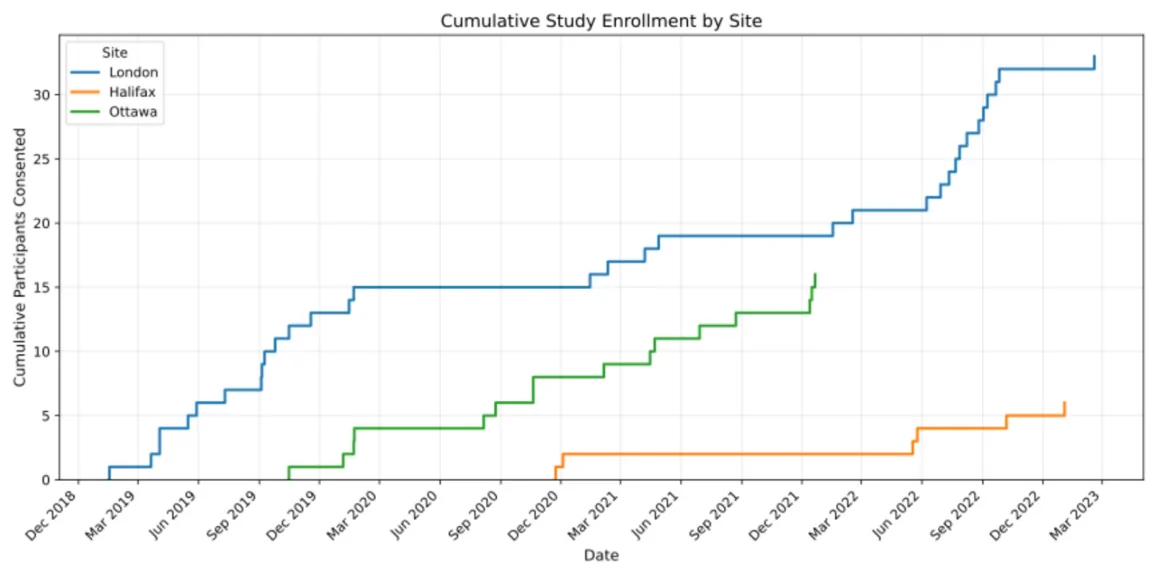
RithMM site-specific recruitment from 2019 to 2023.

Due to the frequent and prolonged social restrictions of health facilities, the timeline of the study had to be adjusted according to each participating site’s capability, leading to a significant decrease in the initially pre-planned time for accrual. Pandemic social restrictions staggered enrollment, as illustrated in the site openings with interspaced accrual (Figure 2).

**Figure 3:**
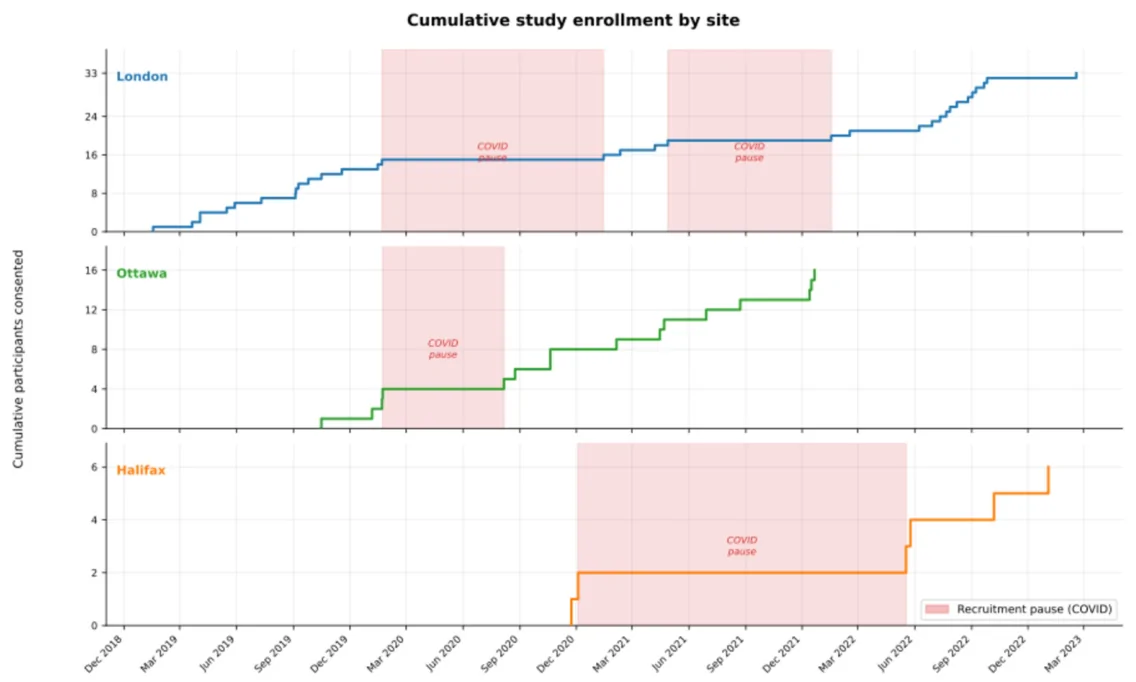
Pauses on active recruitment due to institutional COVID-19 restrictions.

London began recruitment in January 2019, Ottawa began recruitment in October 2019, and Halifax began recruitment in December 2020. After 6 months had passed from the beginning of accrual at the London site, 7 patients had enrolled (1.17 patients per month), Halifax, after 1 month, had 2 (2 patients per month), and Ottawa, after 4 months, enrolled another 4 (1 patient per month). We extended the study timeline due to the delayed study startup at the participating sites within the first 12 months. Throughout the study calendar period, we recruited a total of 55 participants; London recruited 33 participants in 49 months (0.67 participants per month), Ottawa recruited 16 participants in 26.2 months (0.61 participants per month), and Halifax recruited six participants in 25.3 months (0.24 participants per month) (Table 3), not meeting our feasibility goal. However, due to the COVID pandemic, there were multiple site-specific restrictions that limited active recruitment (Figure 3, Table A1). Herein, we present an exploratory post hoc analysis of participant enrollment through *active recruitment time* instead of calendar time. London recruited a total of 33 participants throughout three active recruitment periods, totalling 28.7 months of active recruiting (1.15 participants per month); Ottawa recruited a total of 16 participants in two recruitment periods, totalling 19.7 months of active recruiting (0.81 participants per month); Halifax recruited a total of six participants throughout two recruitment periods, a total of 7.9 months of active recruitment (0.76 participants per month) (Table 4). Accounting for social restrictions throughout the total study period, the cumulative recruitment of 55 participants between all three sites was 56.3 months, a rate of 0.98 participants per month.

### 3.3. Intervention and Safety Efficacy Outcomes

Evidence suggests that TE is reduced in patients with MM regularly taking DOACs, and therefore, we evaluated the safety and efficacy outcomes of ASA and rivaroxaban [16,17]. Secondary efficacy and safety outcomes are depicted in Table 5, Table 6 and Table 7. In the ASA arm, there were two recorded bleeding events (7.1%, 95% CI, 0.9–23.5%); two VTE (7.1%, 95% CI, 0.9–23.5%); and no ATE. One major bleeding event was a gastrointestinal bleed requiring emergent care and hospital admittance, and the other was a CRNMB on the left great toe not requiring hospital admittance. The reported VTEs include one instance of deep vein thrombosis in the right leg, and the other was an instance of pulmonary embolism. The rivaroxaban arm had no bleeding events, VTE or ATE. No minor bleeding events were recorded in the two study arms. During the study, two participants died. One deceased participant in the ASA arm had developed a neck mass with no longer effective chemotherapy/radiation therapy and opted for medically assisted dying services. The second deceased participant was in the rivaroxaban arm and had an acute coronary event approximately one month after completion of trial medication and opted for comfort measures.

### 3.4. Biochemical Predictors of Thrombosis

Collecting data for the potential hypercoagulability predictors was a challenge across the three study sites. There was greater attrition in data collection, specifically at follow-up visit 3 and the end of treatment (90.9% attendance at month 1, 74.5% attendance at month 3, and 76.4% attendance at the end of treatment). However, not all biochemical predictors were consistently measured; therefore, the number of samples collected was not sufficient for exploratory data analysis of trend over time.

**Figure 4:**
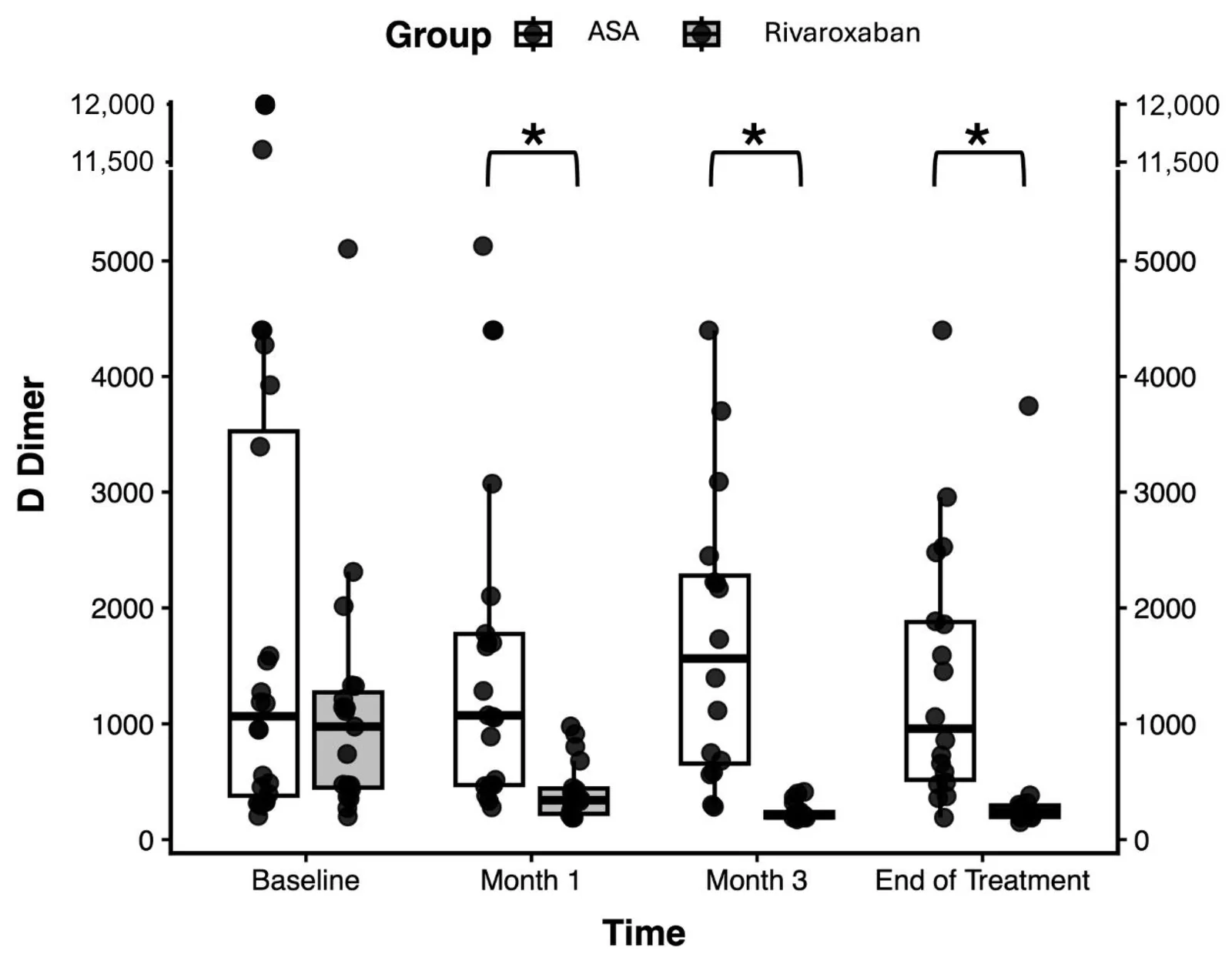
Longitudinal analysis of D-Dimer between the ASA and rivaroxaban across baseline, month 1, month 3, and end of treatment timepoints. Statistical significance (Mann–Whitney U test, *p* < 0.0001) is denoted with an asterisk.

Four biochemical markers were explored for their relation to TE and response to anticoagulants (Table A2). There were no differences between the ASA and rivaroxaban cohorts for LDH, creatinine clearance, and CRP. D-dimer was statistically higher in ASA participants compared to rivaroxaban participants at timepoints month 1, month 3, and end of treatment (Figure 4).

## 4. Discussion

The RithMM trial sought to evaluate the feasibility of conducting a full-scale study involving myeloma patients on lenalidomide/dexamethasone-based therapy to test the safety and efficacy of ASA and rivaroxaban. The individual feasibility parameters defined per site and per month were not met when assessing the time of active enrollment, except in the case of the London site (1.15/month), possibly due to the limitations of health facilities’ closure for non-essential research during COVID-19 public health social restrictions. In the case of London, most participants were accrued before the lockdown, which justifies this site’s better performance. Nevertheless, the original target of enrolling 86 participants across three sites within 12 months was ambitious. Even before the pandemic, London’s recruitment rate was insufficient to support the assumption that this target would have been achieved in the absence of pandemic-related interruptions. Additionally, the complete availability of recruitment was difficult to assess despite defined periods of enrollment pauses, as periods of time outside defined COVID-19 restrictions still involved health advisories encouraging citizens to reduce social interactions along with added cuts in research personnel, which may have negatively impacted trial procedures, especially start-up procedures. For example, despite the London recruitment pause occurring exclusively in 2020, recruitment rates remained low in 2021 and early 2022—a reflection of the third and fourth waves of COVID cases in Canada. These effects are not surprising, as the COVID-19 pandemic caused widespread challenges to clinical research worldwide [35,36,37]. Enrollment was particularly challenging during the COVID-19 pandemic, with alternative techniques for recruitment and informed consent being encouraged, such as techniques for e-consent and Facebook groups for recruitment [38,39]. Canada was no exception to these difficulties, as cancer centre researchers found that patient recruitment and trial site activation in 2020 decreased 67.5% and 81%, respectively, compared to 2019 [40]. When examining the London site after the COVID restrictions, there was a significant increase in enrollment between mid- and late-2022. Nevertheless, we did not enroll the pre-specified 86 participants, but 55 participants. For these reasons, we believe that, had the COVID-19 public health social restrictions not occurred, we may have achieved the pre-specified target. The rates of enrollment during active recruitment periods suggest the feasibility of the full-scale trial, particularly at the London site. With the unanticipated extended duration of the study and decreasing availability of resources, the planning committee decided it was most appropriate to close the study prematurely before reaching the 86-participant goal. For our projection of a full-scale trial with a total participant goal of 398 (199 in each group) participants to demonstrate statistical power, we estimate that with seven participating centres (57 participants per centre at a rate of 0.98 participants per month), it would take a maximum of 58.1 months (4.84 years) to recruit all participants, provided no further recruitment interruptions of any kind; however, if research limitations similar to COVID-19 occur, it would take approximately 103.6 calendar months (8.6 years at a rate of 0.55 participants per month per centre) to recruit sufficient participants and this study should not be considered feasible. Considering the unique challenges during this pilot, we anticipate that recruitment in a full-scale RithMM trial would be greater than 0.98 participants per month. The original sample-size calculation for the full trial assumes a 10% attrition. However, the observed attrition in the pilot was substantially higher: 25.5%. Of total attrition cases, non-compliance and withdrawal were 3.6% each and align with our assumption of a 90% compliance rate. However, we did not anticipate loss of eligibility, the physician’s discretion to stop medication, or administrative error, among others. The combination of these variables was greater than predicted and highlights the need to better anticipate accrual limitations in a future study. For the full trial we will have to account for these causes of attrition as well as try to establish ways to avoid or mitigate some of them before we commit to a sample size for a full-scale trial and consider if it is, in fact, feasible. We have calculated alternative sample sizes for the full-scale trial, summarized in Table A3 and Table A4.

Medication compliance was good for the initial month of the study but decreased slightly during the final 12 weeks of the trial from 87.3% to 69.1%. This was expected, as ASA and rivaroxaban are easily administered as oral tablets. We acknowledge that the increased frequency of virtual care during the COVID-19 pandemic may have contributed to participation disposition, as this study required consistent in-person sampling and follow-up appointments. A study on virtual care at a healthcare centre in Toronto, Canada, found that, of the hematologic malignancies analyzed, patients with myeloma had the highest proportion of virtual care during the pandemic (45.8%) [41]. Overall attrition rates were higher than anticipated, with 14 patients (25.5%) stopping study activities. Participant ineligibility was the most common reason for attrition and only observed in the ASA arm with three participants (5.4%). A full RithMM trial would also monitor participant eligibility closely for health status changes that may impact participation.

There were two participants who were non-compliant with their study drug, both of which were in the rivaroxaban arm. Considering that both medications are easily administered and have a similarly low side effect risk [42], we do not attribute the non-compliance to any safety risk of the study intervention. During the study trial period, one participant died in each arm, for a total of two participants. Neither of these deaths is attributed to the study intervention and the study remains a low-risk clinical trial.

To date, apixaban has been the most widely evaluated anticoagulant in MM [17,43,44]. Our efficacy and safety results with rivaroxaban are in keeping with the current literature in anticoagulation prophylaxis of patients with MM on IMiD-based therapy, which favours the use of DOACs. In the retrospective cohort study by Storrar et al., 70 patients received apixaban 2.5 mg every 12 h for a median of 3.8 months while on lenalidomide or thalidomide for a median of 3.6 months. There were no cases of VTE; two patients (2.9%) had ATE and one (1.4%); had major bleeding [17]. Sayar and colleagues found no thrombotic events in 147 patients with MM on IMiD-based therapy and a 1.2% major bleeding rate for those on apixaban thromboprophylaxis [44]. In Myelaxat, a prospective phase two pilot study, 104 patients with MM on IMiD-based therapy received prophylactic apixaban [45]. There were two (1.9%) VTEs, one major bleeding episode, and 10 (9.6%) CRNMB. Piedra et al. retrospectively investigated the efficacy of rivaroxaban or ASA on patients with MM receiving Len/Dex-based therapy. Amongst three groups of MM combination therapy (RVd-ASA, KRd-ASA, KRd-Riva), there were 305 patients. VTE occurred in 4.8% (6/124) of those on RVd, 16.1% (16/99) of those on KRd-ASA and 4.8% (4/82) in KRd-riva. No major bleeding events were recorded [46]. In our study, we did not collect enough data on biochemical markers to perform a statistical risk analysis, as well as an analysis of biochemical markers between the two cohorts. However, we did measure a statistical significance in D-dimer between ASA and rivaroxaban. This is expected, as other studies have shown a decrease in D-dimer with rivaroxaban, such as a study comparing ASA to ASA + rivaroxaban in patients with diabetes [47] or in patients taking one to two tablets of rivaroxaban suspected to have DVT [48]. This is expected, as D-dimer is a byproduct of fibrin in clots, and rivaroxaban reduces clotting by binding to Factor Xa. However, it is worth noting that D-dimer should be measured before ingesting rivaroxaban, as it can increase false negatives as a diagnostic tool of DVT [48].

Our study has several strengths and limitations. This is the only RCT, to the author’s knowledge, comparing the standard of care low-dose ASA with a DOAC in patients with MM. In addition, our study focused solely on Len/Dex-based therapy, in contrast to other studies including other forms of IMiDs. Patients with MM are subject to polypharmacy because of all active anti-MM therapy and related supportive therapy. Therefore, choosing rivaroxaban for the intervention arm is a strength of our study. Rivaroxaban is a convenient, once-daily oral tablet suitable for ambulatory treatment, even in elderly and frail patients. Moreover, it avoids the logistical challenges, cost and discomfort associated with subcutaneous injection of LMWH.

One limitation of the feasibility aspect of our study is the impact of the accrual of patients due to the closure of research facilities during the COVID-19 pandemic. The enrollment period was prolonged during multiple periods beyond initial expectations due to the pandemic social restrictions, which introduced delays that would not have been present otherwise. In addition, research start-up processes, such as ethics and contractual reviews, were substantially delayed in Ottawa and Halifax, resulting in significant delays in their local study activities compared to London. In addition, we were unable to collect exploratory test samples as pre-specified in our protocol. Considering the increased associated risk of thromboembolic events and COVID-19, it would have been an informative exploratory analysis between both variables; however, we only had two VTE events, and none of them were associated with COVID-19 cases in our study. As a matter of fact, because academic centres restricted non-essential research, we may have missed relevant data collection during this period. Another important limitation is the attrition of 25.5% in study drug compliance. This may have been driven by a few issues in study conduct. In this pilot study, investigators followed participants at three pre-specified study periods after initial assessment (30 days, 90 days and 180 days after accrual). We should consider, in a future study, following patients monthly and assessing medication compliance and adverse events at shorter intervals and trying to mitigate withdrawal and adverse event assessments that may lead to study drug or lenalidomide discontinuation. In addition, we could consider creating an electronic diary to provide participants with daily reminders to take their pills that would also allow participants to confirm medication intake. These limitations will drive the development of new, better, streamlined strategies for sample size determination for our full-scale RithMM trial. Finally, even though our clinical outcomes were excellent, they were not powered to show a meaningful difference between rivaroxaban and ASA in this feasibility trial and are described in this manuscript merely as exploratory data. Additionally, follow-up differed between the treatment arms, with a lower proportion of participants in the rivaroxaban arm completing six-month follow-up than in the ASA arm (70.4% vs. 85.7%). This difference limits the comparability of observed bleeding outcomes, as shorter follow-up may have reduced the opportunity to detect bleeding events in the rivaroxaban arm. Therefore, the absence of observed bleeding events in this arm should be interpreted cautiously. However, the excellent efficacy and safety trend of both ASA and rivaroxaban in our study suggests that these two comparators can be further investigated in the full-scale trial.

In summary, we were able to demonstrate that the methodology of the RithMM feasibility trial is conductable, although we did not successfully reach our pre-defined feasibility goals of 86 participants in 12 months. Recognizing the unforeseen and complicated external challenges with enrollment unique to each participating site, while considering techniques to improve participant recruitment and retention, we believe that there is potential to scale to a full-size, multi-centre, randomized clinical trial, with a powered sample size to compare the risk of ATE/VTE with two different thromboprophylactic strategies. We acknowledge that our secondary objective did not have the power to demonstrate a difference in TE risk between ASA vs. rivaroxaban; however, we did observe major bleeding exclusively in the ASA arm. This comparison warrants further investigation to fulfill this unmet gap in supportive therapy for myeloma patients on Len-Dex-based therapy.

## Figures and Tables

**Table 1:** Participant characteristics and medical history (n = 55).

Characteristic		ASA		Rivaroxaban		Total	
Weight (kg)	Mean	76.43		80.837		78.593	
	(SD)	(23.649)		(19.14)		(21.47)	
Age at baseline	Mean	72.391		72.831		72.607	
	(SD)	(16.633)		(10.951)		(14.004)	
		(N = 28)	Percent	(N = 27)	Percent	(N = 55)	Percent
	≤65	4	14.30%	5	18.50%	9	16.40%
	>65	24	85.70%	22	81.50%	46	83.60%
Site	London	17	60.70%	16	59.30%	33	60%
	Halifax	4	14.30%	2	7.40%	6	10.90%
	Ottawa	7	25%	9	33.30%	16	29.10%
Sex	Female	12	42.90%	9	33.30%	21	38.20%
	Male	16	57.10%	18	66.70%	34	61.80%
Ethnicity	Hispanic or Latino	1	3.60%	1	3.70%	2	3.70%
	Not Hispanic or Latino	26	92.90%	24	88.90%	50	90.91%
	Not Reported	1	3.60%	2	7.40%	3	5.45%
Race	Aboriginal	0	0%	0	0%	0	0%
	Asian	2	7.14%	0	0%	2	3.64%
	Black or African American	0	0%	0	0%	0	0%
	Oceania or other Pacific Islander	0	0%	0	0%	0	0%
	White	24	85.71%	23	85.19%	47	85.50%
	Not Reported	0	0%	1	3.70%	1	1.82%
	Unknown	2	7.14%	3	11.10%	5	9.10%
Stage of Myeloma (ISS)	Stage I	14	50%	11	40.70%	25	45.50%
	Stage II	6	21.40%	11	40.70%	17	30.90%
	Stage III	8	28.60%	5	18.50%	13	23.60%
Bleeding Index Score							
Aged 65 or older		24	85.71%	22	81.48%	46	83.64%
History of GI tract Bleeding		3	10.70%	0	0%	3	5.50%
History of cardiovascular disease		5	17.90%	5	18.50%	10	18.20%
Prior MI, NCT < 33, Diabetes Mellitus, Creatinine >150 µmol/L		10	35.71%	5	18.52%	15	27.27
Bleeding score category	Low risk	4	14.30%	7	25.90%	11	20%
	Intermediate	21	75%	18	66.70%	39	70.90%
	High risk	3	10.70%	2	7.40%	5	9.10%
Current Line of Therapy	First	13	46.40%	13	48.10%	26	47.30%
	Second or More	15	53.60%	14	51.90%	29	52.70%
Lenalidomide dose	<25 mg	19	67.90%	17	63%	36	65.50%
	25 mg	9	32.10%	10	37%	19	34.50%
Dexamethasone dose *	<20 mg	9	32.10%	5	18.50%	14	25.50%
	20 mg	16	57.10%	17	63%	33	60%
	40 mg	3	10.70%	5	18.50%	8	14.50%

* In most cases, dexamethasone was dosed weekly, with some exceptions (n = 6).

**Table 2:** Summary of participant adherence to anticoagulant regimen and study retention.

Anticoagulant Regimen Compliance (%)		ASA		Rivaroxaban		Total	
		(N = 28)	Percent	(N = 27)	Percent	(N = 55)	Percent
1 month	100	26	92.9%	22	81.5%	48	87.3%
	75–99	1	3.6%	1	3.7%	2	3.6%
	50–74	0	0%	0	0%	0	0%
	25–49	0	0%	0	0%	0	0%
	0–24	0	0%	0	0%	0	0%
	Not attended	1	3.6%	4	14.8%	5	9.1%
3 months	100	20	71.4%	19	70.4%	39	70.9%
	75–99	1	3.6%	1	3.7%	2	3.6%
	50–74	0	0%	0	0%	0	0%
	25–49	0	0%	0	0%	0	0%
	0–24	0	0%	0	0%	0	0%
	Not attended	7	25%	7	25.9%	14	25.5%
6 months (end)	100	22	78.6%	16	59.3%	38	69.1%
	75–99	1	3.6%	2	7.4%	3	5.5%
	50–74	0	0%	0	0%	0	0%
	25–49	0	0%	0	0%	0	0%
	0–24	1	3.6%	0	0%	1	1.8%
	Not attended	4	14.3%	9	33.3%	13	23.6%
Participant Disposition	Intervention Retention	21	75%	20	74.0%	41	74.5%
	Intervention Attrition	7	25%	7	25.9%	14	25.5%
	Adverse event	*2*	*7.1*%	*0*	*0*%	*2*	*3.6*%
	Patient withdrawal	*1*	3.6%	*1*	3.7%	*2*	*3.6*%
	Physician discretion	*0*	*0*%	*1*	3.7%	*1*	*1.8*%
	Death	*1*	3.6%	*1*	3.7%	*2*	*3.6*%
	Noncompliance	*0*	*0*%	*2*	*7.4*%	*2*	*3.6*%
	Administrative error	*0*	*0*%	*1*	3.7%	*1*	*1.8*%
	No longer eligible	*3*	*10.7*%	*0*	0%	*3*	*5.4*%
	Unreported	*0*	*0*%	*1*	3.7%	*1*	*1.8*%
Last point of follow up	Baseline	1	3.6%	4	14.8%	5	9.1%
	1 Month	3	10.7%	2	7.4%	5	9.1%
	3 Months	0	0%	2	7.4%	2	3.6%
	6 Months (end)	24	85.7%	19	70.4%	43	78.2%

**Table 3:** Summary of total calendar recruiting time and recruitment rates per centre.

	London	Ottawa	Halifax	Total
Recruitment period	49.0	26.2	25.3	100.5
Total participants	33	16	6	55
**Participants per month**	**0.67**	**0.61**	**0.24**	**0.55**

**Table 4:** Summary of active recruiting time and recruitment rates per centre.

	London	Ottawa	Halifax	Total
Recruitment period 1 (months)	12.2	3.2	0.4	15.8
Recruitment period 2 (months)	3.5	16.5	7.5	27.5
Recruitment period 3 (months)	13.0	N/A	N/A	13
Cumulative recruitment (months)	28.7	19.7	7.9	56.3
Total participants	33	16	6	55
**Participants per month (active recruitment)**	**1.15**	**0.81**	**0.76**	**0.98**

**Table 5:** Incidence of serious adverse events and bleeding episodes.

	ASA (*N* = 28; Total 12.08 Person-Years)				Rivaroxaban (*N* = 27; Total 10.46 Person-Years)				Total (*N* = 55; 22.5 Person-Years)			
Event	Participants with Event (*n*)	Percent	Total Events (*n*)	Event per 1000 Person-Years	Participants with Event (*n*)	Percent	Total Events (*n*)	Event per 1000 Person-Years	Participants with Event (*n*)	Percent	Total Events (*n*)	Event per 1000 Person-Years
Major Bleeding + CRNMB	2	7.10%	2	165.6	0	0%	0	0	2	3.60%	2	88.7
ATE	0	0%	0	0	0	0%	0	0	0	0%	0	0
VTE	2	7.10%	2	165.6	0	0%	0	0	2	3.60%	2	88.7
Death	1	3.60%	1	82.8	1	3.70%	1	95.6	2	3.60%	2	88.7

**Table 6:** Incidence proportion of bleeding episodes and death.

	ASA (*N* = 28; Total 12.08 Person-Years)				Rivaroxaban (*N* = 27; Total 10.46 Person-Years)				Total (*N* = 55; 22.55 Person-Years)			
	Case	Event per Person Year	6-Month Incidence Proportion	95% CI	Case	Event per Person Year	6-Month Incidence Proportion	95% CI	Case	Event per Person Year	6-Month Incidence Proportion	95% CI
Bleeding	2	165.6	7.1%	(0.9, 23.5%)	0	0	0	-	2	88.7	3.6%	(0.4, 12.5%)
ATE	0	0	0		0	0	0	-	0	0	0	-
VTE	2	165.6	7.1%	(0.9, 23.5%)	0	0	0	-	2	88.7	3.6%	(0.4, 12.5%)
Death	1	82.8	3.6%	(0.1, 18.3%)	1	95.6	3.7%	(0.1, 19.0%)	2	88.7	3.6%	(0.4, 12.5%)

**Table 7:** Incidence rate of bleeding episodes and death.

	ASA (*N* = 28; Total 12.08 Person-Years)			Rivaroxaban (*N* = 27; Total 10.46 Person-Years)			Total (*N* = 55; 22.55 Person-Years)		
	Case	6-Month Cumulative Incidence Rate	95% CI	Case	6-Month Cumulative Incidence Rate	95% CI	Case	6-Month Cumulative Incidence	95% CI
Bleeding	2	7.94%	(1.00, 25.85%)	0	0	(0, 16.17%)	2	4.34%	(0.54, 14.80%)
ATE	0	0.00%	(0, 14.16%)	0	0	(0, 16.17%)	0	0	(0, 7.85%)
VTE	2	7.94%	(1.00, 25.85%)	0	0	(0, 16.17%)	2	4.34%	(0.54, 14.80%)
Death	1	4.05%%	(0.10, 20.60%)	1	4.67%	(0.12, 23.38%)	2	4.34%	(0.54, 14.80%)

## Data Availability

The raw data supporting the conclusions of this article will be made available by the authors on request.

## Abbreviations

The following abbreviations are used in this manuscript:

ALT	Alanine aminotransferase
ASA	Acetylsalicylic acid
ATE	Arterial thromboembolism
ATE/VTE	Arterial thromboembolism/venous thromboembolism
CI	Confidence interval
CRNMB	Clinically relevant non-major bleeding
CRP	C-reactive protein
CYP	Cytochrome P450
DOAC	Direct oral anticoagulant
DVT	Deep vein thrombosis
GI	Gastrointestinal
IMiD	Immunomodulatory drug
IMWG	International Myeloma Working Group
KRd	Carfilzomib, lenalidomide, dexamethasone
LDH	Lactate dehydrogenase
LMWH	Low molecular weight heparin
MM	Multiple myeloma
NDMM	Newly diagnosed multiple myeloma
PE	Pulmonary embolism
RCT	Randomized controlled trial
Rd	Lenalidomide, dexamethasone
RRMM	Relapsed/refractory multiple myeloma
RVd	Bortezomib, lenalidomide, dexamethasone
SD	Standard deviation
STATA	Statistical software (STATA)
TE	Thromboembolism
ULN	Upper limit of normal
VTE	Venous thromboembolism

## Appendix A

**Table A1:** Date ranges for active periods of recruitment for each centre.

Centre	Start Period 1	Stop Period 1	Start Period 2	Stop Period 2	Start Period 3	Stop Period 3
London	2019-01-17	2020-01-22	2021-01-14	2021-04-28	2022-01-17	2023-02-17
Halifax	2020-11-23	2020-12-04	2022-05-18	2023-01-03	N/A	N/A
Ottawa	2019-10-16	2020-01-23	2020-08-06	2021-12-21	N/A	N/A

**Table A2:** Laboratory markers of thromboembolic risk.

Characteristic	LDH		Creatinine Clearance		D-Dimer Value		C-Reactive Protein	
	ASA	Riva	ASA	Riva	ASA	Riva	ASA	Riva
	Baseline							
Mean	187.42	175.09	71.92	75.10	2347.00	1128.8	6.18	3.89
Standard Deviation	80.95	35.65	37.97	37.91	3248.95	1125.6	8.06	6.81
Range	387.00	141.00	186.14	143.08	11,787.00	4904.0	35.50	34.70
Median	173.50	177.50	71.19	65.00	1064.5	976.00	3.10	2.25
Count	24.00	22.00	28.00	27.00	24.00	19.00	27.00	24.00
	Month 1							
Mean	194.72	183.83	68.50	73.53	1629.2	421.11	12.64	7.46
Standard Deviation	62.36	40.26	36.63	29.68	1447.6	251.91	21.79	10.34
Range	274.00	264.00	170.00	136.09	4849.0	977.00	76.00	39.40
Median	186.00	186.00	64.45	76.71	1073.0	339.50	4.60	3.90
Count	25.00	23.00	26.00	22.00	21.00	18.00	23.00	19.00
	Month 3							
Mean	190.36	200.71	73.88	74.64	1727.6	242.88	7.95	6.40
Standard Deviation	45.01	30.23	37.51	33.66	1249.4	78.35	10.36	11.93
Range	173.00	99.00	152.60	137.35	4115.0	236.00	36.40	50.50
Median	198.50	200.00	62.00	73.10	1563.5	208.00	3.60	3.00
Count	22.00	17.00	21.00	20.00	16.00	17.00	18.00	18.00
	End of Treatment							
Mean	188.50	198.17	72.11	78.78	1385.2	491.86	12.42	3.94
Standard Deviation	58.24	51.81	36.77	36.22	1129.4	938.34	30.08	5.02
Range	231.00	216.00	146.08	138.90	4209.0	3594.00	131.70	19.60
Median	181.00	198.00	63.00	72.00	958.00	238.50	1.50	2.70
Count	20.00	18.00	21.00	19.00	18.00	14.00	19.00	15.00

**Table A3:** Full-scale RithMM trial sample size calculations.

Projection	Power	ASA TE Risk	RIVA TE Risk	Fleiss: ASA	Fleiss: RIVA	Fleiss CC: ASA	Fleiss CC: RIVA	Total Fleiss	Total Fleiss CC
1	80%	5%	2%	594	594	659	659	1188	1318
2	80%	10%	2%	136	136	160	160	272	320
3	90%	10%	2%	181	181	205	205	362	410
4	80%	8%	2%	203	203	235	235	406	470
5	80%	8%	4%	555	555	604	604	1110	1208
6	80%	10%	4%	289	289	322	322	578	644

**Table A4:** Full trial: attrition rates calculations to add to sample size required for 90% power and 10% TE risk for ASA and 2% for RIVA.

Attrition Rate	0%	10%	15%	20%	25%
Calculated N	362	402	426	453	483
To have equal number of N in each arm	362	402	426	454	484
